# Modification and optimization of electrospun gelatin sheets by electron beam irradiation for soft tissue engineering

**DOI:** 10.1186/s40824-017-0100-z

**Published:** 2017-07-11

**Authors:** Jae Baek Lee, Young-Gwang Ko, Donghwan Cho, Won Ho Park, Oh Hyeong Kwon

**Affiliations:** 1 Department of Polymer Science and Engineering, Kumoh National Institute of Technology, Gumi, Gyeongbuk 39177 Korea; 2 Department of Advanced Organic Materials and Textile System Engineering, Chungnam National University, Daejeon, 34134 Korea

**Keywords:** Gelatin, Electrospinning, Nanofiber, Electron beam, Biodegradation

## Abstract

**Background:**

Crosslinked gelatin nanofibers are one of the widely used scaffolds for soft tissue engineering. However, modifying the biodegradation rate of chemically crosslinked gelatin is necessary to facilitate cell migration and tissue regeneration. Here, we investigated the optimal electron beam (e-beam) irradiation doses with biodegradation behavior on changes in the molecular weight, morphology, pore structure, and cell proliferation profiles of electrospun nanofibrous gelatin sheets.

**Methods:**

The molecular weights of uncrosslinked gelatin nanofibers were measured using gel permeation chromatography. The morphology and pore structure of the gelatin scaffolds were analyzed by scanning electron microscopy and a porosimeter. Biodegradation tests were performed in phosphate-buffered saline solutions for 4 weeks. Cell proliferation and tissue regeneration profiles were examined in fibroblasts using WST-1 assays and hematoxylin and eosin staining.

**Results:**

Crosslinked gelatin nanofiber sheets exposed to e-beam irradiation over 300 kGy showed approximately 50% weight loss in 2 weeks. Gelatin scaffolds exposed to e-beam irradiation at 100–200 kGy showed significantly increased cell proliferation after 7 days of incubation.

**Conclusions:**

These findings suggested that the biodegradation and cell proliferation rates of gelatin nanofiber scaffolds could be optimized by varying e-beam irradiation doses for soft tissue engineering.

## Background

Gelatin, a protein derived from controlled hydrolysis of collagen, is potentially useful as a biomaterial because of its biological origin, biocompatibility, non-antigenicity, and biodegradability. Furthermore, the existence of unique sequences of amino acids and many functional side groups makes it easy to modulate its physicochemical properties [1–3]; thus, gelatin has been widely used as tissue engineering scaffold, drug delivery carrier, wound dressing, and sealant for vascular prostheses in the biomedical field. However, gelatin must be modified for use in biomedical applications because it is soluble in aqueous solution and shows mechanical weakness. The typical technique for gelatin modification is crosslinking with a variety of chemical and physical treatments [1, 4–8]. Researchers have studied various crosslinking methods, including chemical crosslinking with glutaraldehyde, 1-ethyl-3-(3-dimethylamino propyl) carbodiimide hydrochloride (EDC), and genipin and physical crosslinking (e.g., dehydrothermal treatment or exposure to plasma, UV, and γ-radiation). Generally, chemical methods have high efficiency for stabilization of crosslinked gelatin; however, the degree of transformation cannot be controlled precisely [9, 10]. Furthermore, highly crosslinked gelatin materials are not suitable for use in vitro because they are rarely hydrolyzed without enzyme. In contrast, physical methods provide cost-efficient and environmentally friendly alternatives, but yield a lower degree of crosslinking. Mediation of the crosslinking type for gelatin is necessary to achieve desirable properties for particular biomedical applications.

Among reported physical crosslinking methods, ionizing irradiation treatment with γ-ray or electron beam (e-beam) radiation is operationally fast and does not require additives or severe conditions, which could deform the material structure [11–20]. Moreover, the high energy of irradiation can cause both crosslinking and degradation of the material and alter the physical and chemical characteristics of the material through main chain scission and crosslinking. After irradiation, excited polymers, such as reactive intermediates, ions, and free radicals, are formed, and these active species create new bonds and alter material properties as a result of initiation or free reaction among the polymer chain [16, 21, 22]. Crosslinking occurs when two radicals induced by irradiation combine to form a covalent bond, leading to an increase in molecular weight. In contrast, degradation arises out of high energy in excess of the attractive forces between atoms, thus rupturing the chemical bonds and causing a decrease in molecular weight. As a result of these reactions, ionizing radiation is related to the lifespan of the materials via crosslinking and chain scission.

Although some studies have assessed the effects of γ-radiation on gelatin, very few studies have investigated the effects of e-beam irradiation on gelatin [12–17]. Accordingly, in this study, we focused on the effects of e-beam irradiation on changes in the molecular weight, morphology, and biodegradation rate of nanofibrous gelatin sheet scaffolds. The primary objective of this study is to assess the e-beam irradiation dosage on gelatin nanofiber sheets in order to optimize the biodegradation rate for use as a soft tissue engineering scaffold.

## Methods

### Electrospinning of gelatin nanofibers

Type B gelatin (250 bloom) was obtained from Geltech Co., Korea, and 2,2,2-trifluoroethanol (TFE) was purchased from Sigma-Aldrich (USA). Gelatin nanofiber sheets were fabricated using electrospinning of a gelatin-TFE solution. Gelatin was dissolved in TFE at 10% (*w*/*v*) by stirring overnight at room temperature. Electrospinning was performed using a 10-mL polypropylene syringe with a 21-gauge needle. A high voltage of 15 kV was applied to the needle and stainless steel drum collector (drum diameter: 23 cm). The distance between the needle and collector was set as 12 cm. The flow rate of the gelatin-TFE solution was 2 mL/h. The electrospun sheets were placed in a vacuum oven overnight at room temperature to remove a residual solvent.

### Crosslinking of nanofibrous gelatin sheets

Gelatin nanofiber sheets were crosslinked by glutaraldehyde (25%; Daejung Chemicals & Materials Co., Ltd., Korea). The vapor crosslinking process was carried out for 6 h in a sealed desiccator with 25% glutaraldehyde (10 mL) in a petri dish [5, 6]. Electrospun gelatin sheets were placed on a stainless steel wire net in the desiccator to avoid uneven crosslinking and crosslinked with the glutaraldehyde vapor at 25 °C. After crosslinking, the samples were placed in a vacuum oven for 24 h to prevent excessive crosslinking by removing residual glutaraldehyde. Crosslinked gelatin nanofiber sheets were kept at 4 °C before use.

### E-beam irradiation

E-beam irradiation of the gelatin nanofibrous sheets was performed using an Electron Beam Accelerator (ELV-8; EB Tech Co., Ltd., Korea). Uncrosslinked and crosslinked gelatin samples were irradiated to investigate the effects of e-beam radiation. For uncrosslinked gelatin, irradiation of the e-beam was carried out in an air and N_2_ atmosphere at room temperature. The irradiation doses were 10, 20, 30, 40, 50, 60, 70, 80, 90, 100, 150, 200, 250, and 300 kGy. For crosslinked gelatin, irradiation of the e-beam was performed in air at room temperature. The doses were 100, 200, 300, 400, 500, and 600 kGy. For each treatment, the accelerating voltage of the e-beam energy and current were 1 MeV and 17 mA, respectively, and the dose rate of the e-beam was 8.33 kGy/s.

### Scanning electron microscopy

The surface morphology of crosslinked and uncrosslinked gelatin samples was observed using a scanning electron microscope (SEM; JSM-6380; JEOL, Japan). Crosslinked samples were immersed in purified water and lyophilized to observe the fibrous structure of gelatin. Dry samples of gelatin were observed with 11 kV accelerating voltage after sputter coating with platinum. The diameters of nanofiber strands were determined using an image measurement software (I-solution Lite, IMT i-solution Inc., Korea).

### Pore size and porosity

The pore size and porosity of the gelatin nanofiber sheets were determined by multiple point Brunauer, Emmet and Teller (BET) gas adsorption measurement using NOVA 2000 and Autosorb-1-C instruments (Quantachrome, Japan).

### Measurement of molecular weight

Changes in the molecular weight of each e-beam-irradiated uncrosslinked gelatin sheet were determined using gel permeation chromatography (GPC; Breeze System, Waters, USA) at 40 °C with a refractive index detector. We used 0.02 N NaNO_3_ as a solvent with a flow rate of 0.8 mL/min. The GPC calibration curve was obtained using pullulan standards.

### Quantification of crosslinking degree

The degree of crosslinking was determined using 2,4,6-trinitrobenzensulfonic acid (TNBSA; Thermo Scientific, USA) by calculating the primary amine content of crosslinked and uncrosslinked gelatin samples. Briefly, 2–4 mg of gelatin was placed in a conical tube with 1.0 mL of 4% (*w*/*v*) sodium bicarbonate solution (NaHCO_3_, pH 8.5) and 1.0 mL of 0.5% (*w*/*v*) in methanol. After incubation at 40 °C for 2 h, the solution was treated with 3 mL of 6 M HCl, and the temperature was increased to 60 °C for 1.5 h to solubilize gelatin samples. The absorbance of the resulting solution was measured at 345 nm using a spectrophotometer (Optizen 3220UV; Mecasys Co., Ltd., Korea) after diluting with 4 volumes of deionized water. The degree of crosslinking was calculated using the following equation [9]:

$$ \mathrm{Crosslinking}\ \mathrm{degree}\ \left(\%\right)=\left[1-\left(\frac{absorbance_c/{mass}_c}{absorbance_u/{mass}_u}\right)\right]\times 100 $$

where the subscripts c and u stand for the crosslinked and uncrosslinked gelatin, respectively (*n* = 3).

### Nonenzymatic biodegradation test

Each e-beam-irradiated gelatin sample was cut into dimensions of 20 × 10 mm, and the initial weight of each specimen was measured. The samples placed in conical tubes with 10 mL phosphate-buffered saline (PBS, pH 7.4) and incubated in a shaking water bath (37 °C with shaking at 0.15 m/s) for 1, 2, 3, or 4 weeks. After each designated period, samples were rinsed twice with purified water and lyophilized. Thereafter, the weight after the degradation test was measured, and the percent weight loss was calculated using the following equation:

$$ \mathrm{Weight}\ \mathrm{loss}\ \left(\%\right)=\frac{W_i-{W}_f}{W_i}\times 100 $$

where *W*_*i*_ is the initial weight of the gelatin sheet, and *W*_*f*_ is the weight of the gelatin sheet after the degradation test (*n* = 5).

### Water absorption test

The hydrophilicity of the e-beam irradiated gelatin sheets was evaluated using a contact angle analyzer (Phoenix 300; SEO Co., Ltd., Korea) by measuring the complete water absorption time from a deposited droplet of purified water onto the surface of the irradiated gelatin nanofiber sheets at 25 °C and 50% humidity. The results were expressed as the mean ± standard deviation (*n* = 5).

### Cell proliferation

Fibroblasts (NIH-3 T3 cells) were used to evaluate cell proliferation on e-beam-irradiated gelatin nanofiber scaffolds. Samples were cut into a disk shape (22 mm in diameter), immersed in 70% ethanol for sterilization for 24 h, and then rinsed with PBS for 24 h before seeding. Prepared gelatin fibrous sheets were placed in 12-well culture plates with glass rings to prevent floating. Cells were seeded at a density of 5.0 × 10^4^ cells/disk in 1 mL Dulbecco’s modified Eagle medium (DMEM; Welgene, Korea) containing 10% fetal bovine serum (FBS; Welgene, Korea) and 1% antibiotics (Penicillin-Streptomycin solution; Welgene). Seeded cells were cultured for 1, 3, 5, or 7 days under standard cell culture conditions. After the designated culture period, the culture medium was replaced with 1 mL of medium containing 10% WST-1 reagent. Subsequently, samples were incubated under standard cell culture conditions for 2 h, and 100 μL of the incubated solution was then transferred to a 96-well culture plate. A microplate reader (PHOmo; Autobio Labtec Instruments Co., Ltd., China) was used to measure the absorbance of formazan at 450 nm.

### Histological analysis

Nonirradiated and irradiated (200 kGy) crosslinked gelatin scaffolds were used for tissue culture experiments at a density of 5.0 × 10^5^ cells/cm^2^ with 15 mL cell culture medium. Cell-seeded gelatin scaffolds were rinsed with medium to eliminate detached cells and incubated for 3 weeks in a CO_2_ incubator, with daily changes in culture medium. After incubation, the tissue-scaffold constructs were fixed in 4% formaldehyde aqueous solution and processed for routine paraffin embedding and sectioning. The cell cultured gelatin scaffold sections were stained with hematoxylin and eosin (H&E) to evaluate migration into the gelatin sheet and observed under an optical microscope (Eclipse TS100; Nikon, Japan) equipped with a digital camera (DS-Fi-2; Nikon, Japan).

### Statistical analysis

Values are expressed as means ± standard deviations. Statistical analysis was performed using Student’s *t*-tests. Results with *p* values of less than 0.05 were considered significant.

## Results

### Morphologies and molecular weights of uncrosslinked gelatin nanofibers

**Fig. 1:**
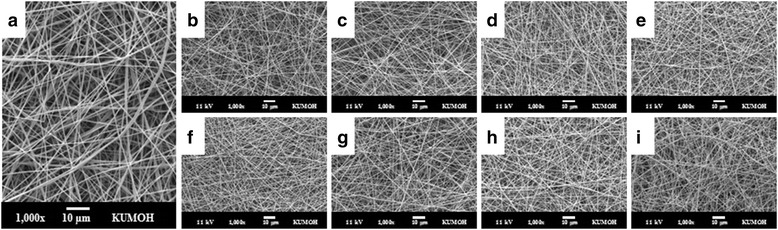
SEM images of uncrosslinked gelatin nanofibers (**a**) and uncrosslinked gelatin nanofibers with e-beam irradiation doses of 10 (**b**), 50 (**c**), 100 (**d**), and 300 kGy (**e**) in air and 10 (**f**), 50 (**g**), 100 (**h**), and 300 kGy (**i**) in N_2_ atmosphere. Scale bars are 10 μm

Electrospun gelatin nanofibers showed an even cylindrical shape with an average fiber diameter of 443 ± 114 nm (Fig. 1a). Since gelatin is a water-soluble material, the structure is easily collapsed under aqueous conditions. Therefore, gelatin is commonly crosslinked for use as a biomaterial with chemical or physical methods. Among the physical methods published to date, e-beam irradiation is considered one of the most efficient ways to modify materials for suitable mechanical and thermal properties. After irradiation, morphological deformation was not observed in all irradiated gelatin nanofibers, regardless of the irradiation dosage or atmosphere (air, N_2_; Fig. 1b–i).

**Fig. 2:**
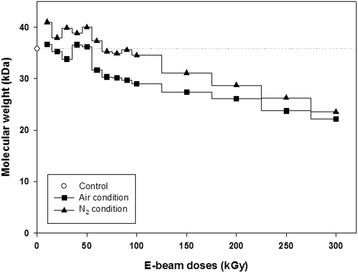
Changes in the molecular weights of uncrosslinked gelatin nanofibers as a function of e-beam irradiation dose in air and N_2_ conditions. The control (35.79 kDa) refers to the molecular weight of gelatin nanofibers before e-beam irradiation

However, changes in the molecular weight (Mw) of gelatin fibers as a function of e-beam irradiation dose were observed (Fig. 2). The molecular weight of gelatin fibers after irradiation at over 60 kGy in an N_2_ atmosphere or at the entire range of irradiation dosages in air gradually decreased in a dose-dependent manner. In contrast, the molecular weights of gelatin nanofibers irradiated at less than 60 kGy in an N_2_ atmosphere were increased in comparison with those of nonirradiated gelatin nanofibers.

### Morphology of crosslinked gelatin nanofibers

**Fig. 3:**
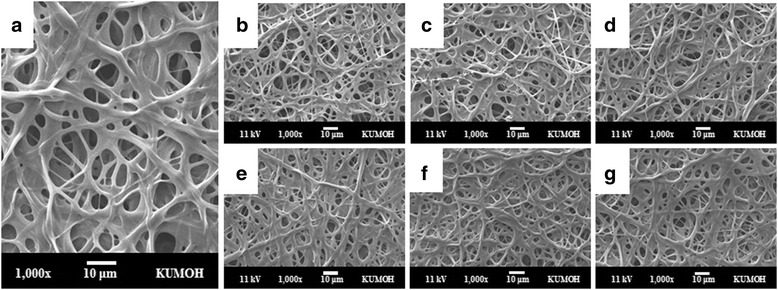
SEM images of the crosslinked gelatin fibrous sheet (**a**) and e-beam-irradiated gelatin sheets with applied doses of 100 (**b**), 200 (**c**), 300 (**d**), 400 (**e**), 500 (**f**), and 600 kGy (**g**). All nanofibers were crosslinked by glutaraldehyde vapor before e-beam irradiation. Scale bars are 10 μm

The microstructures of crosslinked gelatin nanofibers with glutaraldehyde vapor following e-beam irradiation are shown in Fig. 3. The phenomena of partial aggregation and conglutination with each fiber were observed after crosslinking. Pore size was increased from 8.5 to 9.3 μm, and porosity was increased to about 17.7% in crosslinked gelatin sheets (Table 1). These characteristic features in the crosslinked gelatin facilitated cell migration and proliferation. The pore size and porosity in electrospun fibers increased as the fiber diameter increased (Fig 3a). Furthermore, the increased pore size enhanced the cell supporting capacity by increasing cell migration and nutrient flow into the scaffold and appeared the most favorable scaffold in vitro, indicating the occurrence of cell infiltration at seeding, cell viability, and optimal cell organization. Additionally, porosity should be as high as 90% to ensure nutrient flow and tissue regeneration. In this study, we achieved 88.4% porosity in the crosslinked gelatin, which was suitable for application as a scaffold; this high porosity indicated that the crosslinked gelatin maintained an interconnected pore structure.

**Table 1:** Structural properties of fibrous gelatin sheets after crosslinking with glutaraldehyde vapor (*n* = 50)

Samples	Fiber diameter (nm)	Pore size (μm)	Porosity (%)
Uncrosslinked gelatin sheet	443 ± 101	8.5	70.7
Crosslinked gelatin sheet	2069 ± 865	9.3	88.4

### Crosslinking degree of gelatin nanofibers

The degree of crosslinking after electron beam irradiation with varied irradiation doses (100, 200, 300, 400, 500, 600 kGy) was, 40 ± 3, 37 ± 3, 35 ± 2, 27 ± 4, 22 ± 5, 16 ± 4%. The degree of crosslinking in nonirradiated crosslinked gelatin was 48 ± 4% and decreased as e-beam irradiation increased, reaching a minimum value of 15.5%. The results indicated that the high energy of e-beams promoted the cleavage of the chemical bonds of gelatin, including the site of crosslinking. However, the morphologies of crosslinked gelatin nanofibers after e-beam irradiation were not significantly different from those of nonirradiated crosslinked gelatin (Fig. 3b–g).

### Biodegradation behavior

**Fig. 4:**
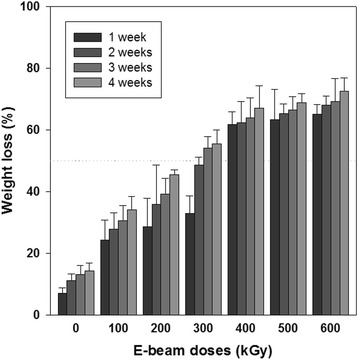
Weight loss of crosslinked gelatin fibrous sheets as a function of irradiated dose and incubation period (PBS, 37 °C; *n* = 5)

Biodegradation behavior as a function of e-beam irradiation in a nonenzymatic aqueous system was determined using irradiated gelatin sheets with increasing radiation dosages. The percent weight loss of crosslinked gelatin sheets irradiated with 100, 200, 300, 400, 500, and 600 kGy after incubation in nonenzymatic PBS solution at 37 °C for 1, 2, 3, or 4 weeks is shown in Fig. 4. Weight loss was increased as the irradiation dosage increased. The crosslinked gelatin sheets irradiated at 600 kGy exhibited the most weight loss (more than 70%), with nearly complete decomposition after 4 weeks. For nonirradiated sheets, however, only 15% weight loss was observed during the same period of incubation.

**Fig. 5:**
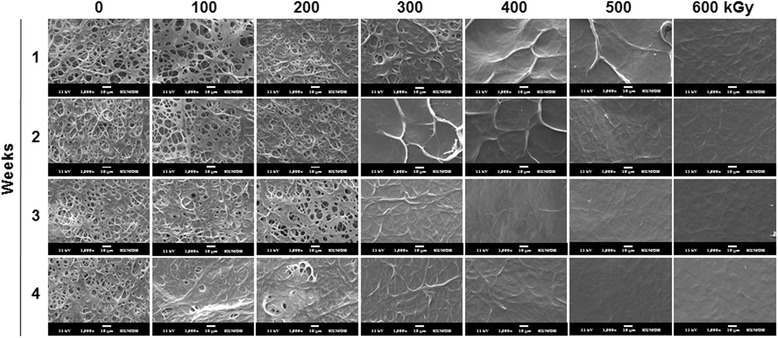
Morphology of crosslinked gelatin fibrous sheets as a function of irradiation dose and incubation period at 37 °C in PBS. Scale bars are 10 μm

Morphology as a function of incubation time is shown in Fig. 5. Rapid degradation on crosslinked gelatin was observed during the early stage in SEM images after irradiation at 400, 500, and 600 kGy. Nonirradiated samples and samples irradiated at doses of 100 and 200 kGy maintained their original fibrous structure for 2 weeks and then slowly degraded over time. In contrast, gelatin sheets irradiated with more than 400 kGy showed collapsing of their fibrous and pore structures after 1 week of incubation. These results showed that e-beam irradiation of crosslinked gelatin accelerated in vitro biodegradation without collagenase.

### Hydrophilicity of e-beam-irradiated gelatin nanofiber sheets

**Fig. 6:**
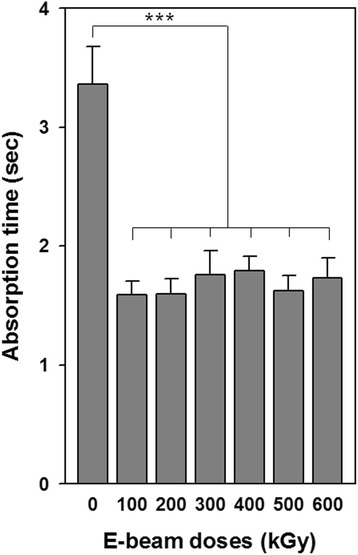
Water absorption of crosslinked gelatin fibrous sheets exposed to varying irradiation doses (water, 25 °C; *n* = 5). ***p < 0.001

To better understand the effects of e-beam irradiation on surface hydrophilic properties, the water absorption rate of nonirradiated and irradiated gelatin sheets was measured on a series of samples with increasing irradiation dosages (Fig. 6). Generally, the equilibrium state of the water droplets on the material reflects the surface properties, such as hydrophilicity, hydrophobicity, and wettability. However, hydrophilic or hygroscopic materials never reach an equilibrium state because the contact angle gradually decreases to 0°. Thus, the water absorption rate was compared between nonirradiated and irradiated samples to determine the hydrophilicity of the samples. The time from the moment of reaching the droplet on a sample to the moment of complete disappearance was measured. Nonirradiated crosslinked gelatin sheets required 3.36 ± 0.32 s to completely absorb water. After irradiation, absorption rates were decreased at all irradiation doses down to 1.59 ± 0.11 to 1.79 ± 0.12 s.

### Cell proliferation on e-beam-irradiated gelatin scaffolds

**Fig. 7:**
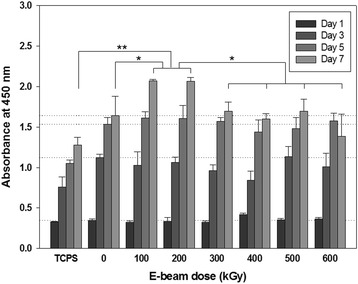
Cell proliferation behaviors on e-beam-irradiated gelatin fibrous sheets. Reference lines indicate the values for the fibrous gelatin sheets (crosslinked without e-beam irradiation) as a control group. *p < 0.05, **p < 0.01

NIH3T3 fibroblasts were cultured on irradiated gelatin sheets to determine the compatibility and proliferation of the cells. WST-1 assays were utilized to evaluate the number of metabolically active cells cultured on e-beam-irradiated gelatin sheets (Fig. 7). Initial cell attachment of nonirradiated and irradiated groups of gelatin sheets did not differ significantly compared with that of tissue culture polystyrene (TCPS). After 3, 5, and 7 days of culture, cell viability on all gelatin sheets was higher than that on TCPS, presumably because gelatin contains integrin-binding sites that enhance cell attachment and promote proliferation. However, differences were not observed as a function of e-beam dosage, except for proliferation after 7 days of culture. Cell proliferation on gelatin samples irradiated at 100 and 200 kGy was significantly higher than that in other groups of irradiated gelatin.

### Tissue cultivation

**Fig. 8:**
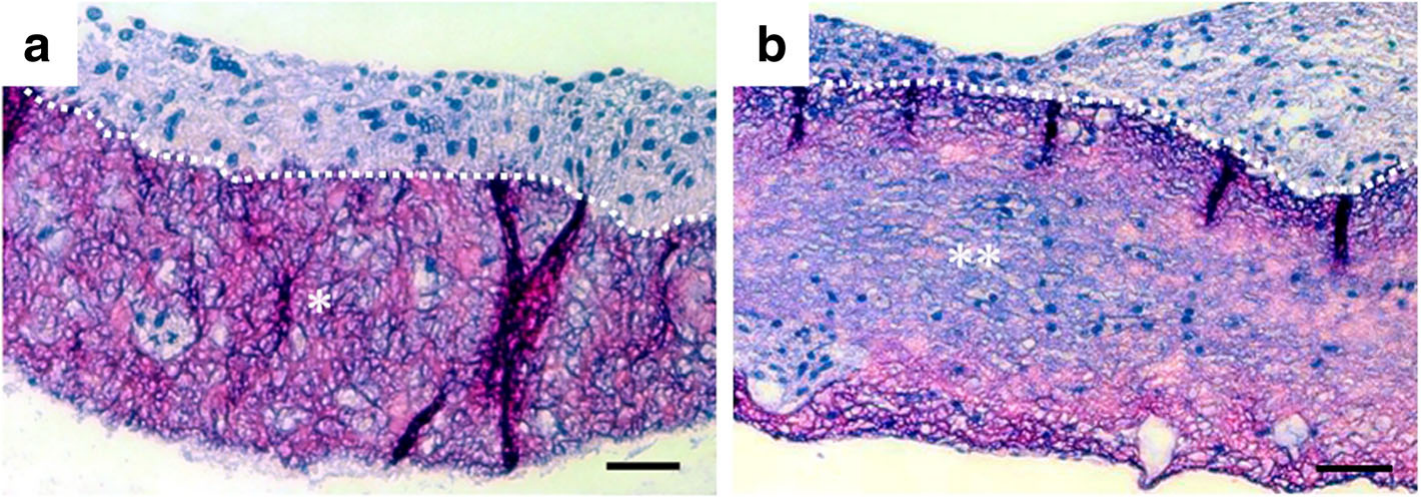
H&E-stained cross-sections of nonirradiated gelatin scaffolds (**a**) and e-beam-irradiated gelatin scaffolds (300 kGy) (**b**) after 4 weeks of fibroblasts culture. Dot lines indicate the top surface boundary of scaffolds. Note the remaining gelatin nanofiber scaffold (*) in the nonirradiated group and the degraded gelatin scaffold (**) in the irradiated group. Scale bars are 50 μm

Histological images of the cell-seeded gelatin nanofiber scaffolds after 4 weeks of culture are shown in Fig. 8. The histological images showed that cultured fibroblasts migrated from the surface of the scaffold into the inner part of the electrospun gelatin and proliferated very well after 4 weeks of culture. The nonirradiated gelatin scaffolds (Fig. 8a) remained the network structure. Otherwise, e-beam-irradiated (300 kGy) gelatin scaffolds (Fig. 8b) was almost degraded compared to nonirradited gelatin scaffold group after 4 weeks of fibroblasts culture. The fibrous gelatin structure gradually disappeared and degraded space was spontaneously occupied by cells.

## Discussion

In this study, we focused on not only nanofibrous morphology of gelatin sheet scaffolds but also degradation of gelatin molecules. For instance, we investigated optimum e-beam radiation for molecular degradation as a gelatin nanofiber sheet scaffold. Electrospun gelatin fibrous sheets treated with e-beam irradiation maintained fibrous networks without morphological deformation for both uncrosslinked and crosslinked gelatin (Figs. 1 and 3). E-beam irradiation could modify polymeric properties by changing the structures of macromolecular chains without microstructural deformation. In the radiation chemical reaction, crosslinking and degradation rate compete with each other, and predominance is determined by the molecular structure, presence of additives (such as initiators and crosslinking agents), and radiation kinetic conditions (such as moisture, temperature, pressure, and radiation conditions). Irradiation of polypeptide-based materials in the solid state generally leads to polymeric chain scission rather than crosslinking because polypeptide structures include many intermolecular forces. However, the molecular weights of gelatin nanofibers irradiated in an N_2_ atmosphere are increased at under 60 kGy (Fig. 2). This result can be explained by the formation of intermolecular networks between gelatin molecules by high-energy e-beams, similar to other crosslinking methods, and these conditions can be used for crosslinking of gelatin materials. However, the modified gelatin nanofibers were dissolved in water (data are not shown), and increases in molecular weight were not sufficient for producing sufficiently modified biomaterials.

Crosslinked gelatin nanofibers enabled modification of the polymeric molecular chain without microstructural deformation by irradiation with e-beams. The degree of crosslinking is mainly correlated with the lifespan of the biomaterials, and highly crosslinked biomaterials can resist both enzymatic degradation and hydrolysis because the degradation rate is generally related to molecular weight and crosslinking density. Accordingly, e-beam irradiation could be an effective method for adjustment of degradation rates in vitro and in vivo. Notably, amide bonds are more resistant to hydrolysis than ester bonds, and biodegradation of crosslinked proteins rarely occurs in vitro since in vitro conditions are different from the conditions observed in vivo. For this reason, scaffolds made of crosslinked proteins can be implanted into animals or can use enzymes for degradation.

In crosslinked gelatin, high irradiation dosages decreased the crosslinking density and affected the biodegradation (Figs. 4 and 5) and hydrophilicity (Fig. 6) of gelatin. Higher irradiation doses applied to crosslinked sheets caused more cleavage of crosslinked gelatin. After irradiation at over 400 kGy, the weight after 1 week was dramatically decreased, and weight loss at 2, 3, and 4 weeks was insignificant. This phenomenon could be responsible for the weight loss of crosslinked sheets through release of short gelatin chains formed after cleavage of chemical bonds by high dosages of e-beam irradiation and exposure to remaining free radicals generated after e-beam treatment.

Changes in water absorption rates were correlated with crosslinking and irradiation. In collagenous materials, previous studies showed that the water absorption ability decreased after crosslinking because the reaction between the ε-amino group and functional group in a crosslinking agent could weaken protonation. As mentioned above, polymeric chain scission and decreased crosslinking after e-beam irradiation could cause increased water absorption ability. Another reason is the direct correlation with e-beam irradiation. After e-beam treatment, the polar component and surface tension of gelatin increased, and this enhancement was related to the decrease in surface hydrophobicity because of reorganization of hydrophilic groups or generation of charged groups on the surface.

The scaffold should keep their physical and chemical structure before cell attachment and proliferation. Then, the scaffolds are degraded according to meet tissue formation. The immediate effect of e-beam irradiation and increased hydrophilicity after e-beam treatment on cell attachment and proliferation were not observed (Fig. 7). However, cultured fibroblasts on gelatin sheets irradiated at 100 and 200 kGy could migrate into sheets and proliferate on both the surface and inner part of the crosslinked gelatin. The structural collapse of gelatin sheets irradiated at more than 300 kGy was determined via microstructural analysis; cells could not migrate into the inner part of the material and proliferated only on the surface. Moreover, noncrosslinked gelatin did not allow proliferation in the inner part compared with irradiated gelatin because the degree of swelling of crosslinked collagenous materials decreased as the crosslinking density increased. Furthermore, irradiated gelatin was easily degraded in PBS.

Migration of proliferated cells into a scaffold is essential for cultivation of soft tissues. To facilitate migration of fibroblasts into the gelatin nanofiber scaffolds, biodegradation should be induced in a week to ensure the inner space of proliferated cells and transportation of nutrients, oxygen and wastes. The gelatin nanofiber scaffold treated with 300 kGy e-beam irradiation showed tissue migration compared to the control scaffold. Based on our SEM, weight loss, cell proliferation and tissue formation results, the 300 kGy e-beam radiation dose demonstrated appropriate condition for soft tissue engineering application.

## Conclusions

Electrospun gelatin fibrous sheets treated with e-beam irradiation maintained fibrous networks without morphological deformation for both uncrosslinked and crosslinked gelatin. The molecular weight data of uncrosslinked gelatin as a function of e-beam irradiation revealed that gelatin nanofibers irradiated at under 60 kGy in an N_2_ atmosphere were crosslinked by high-energy e-beam irradiation. In crosslinked gelatin, high irradiation dosages decreased the crosslinking density and affected the biodegradation and hydrophilicity of gelatin. Physicochemical properties were altered by e-beam irradiation, but the immediate effects of e-beam irradiation on cell attachment and proliferation were not observed. E-beam irradiation could modify the properties of both of uncrosslinked and crosslinked gelatin nanofibrous sheets by changing macromolecular chains without microstructural deformation. In particular, irradiation of crosslinked gelatin resulted in biodegradation without enzyme in PBS, and the lifespan of crosslinked gelatin could be modulated by adjusting the irradiation dosage, demonstrating its potential applications in tissue engineering.
